# Relationship-Based Care and Behaviours of Residents in Long-Term Care Facilities

**DOI:** 10.1155/2014/949180

**Published:** 2014-01-12

**Authors:** Johanne Desrosiers, Anabelle Viau-Guay, Marie Bellemare, Louis Trudel, Isabelle Feillou, Anne-Céline Guyon

**Affiliations:** ^1^School of Rehabilitation, Faculty of Medicine and Health Sciences, Université de Sherbrooke, 3001 12th Avenue North Sherbrooke, Sherbrooke, QC, Canada J1H 5N4; ^2^Research Centre on Aging, CSSS-IUGS, Sherbrooke, QC, Canada; ^3^Département d'études sur l'Enseignement et l'Apprentissage, Université Laval, QC, Canada; ^4^Centre de Recherche et d'Intervention sur la Réussite Scolaire (CRIRES), QC, Canada; ^5^Département des Relations Industrielles, Université Laval, QC, Canada; ^6^Chaire de Recherche en Gestion de la Santé et de la sécurité du travail, Université Laval, QC, Canada; ^7^Département de Réadaptation, Université Laval, QC, Canada

## Abstract

*Introduction*. In long-term care (LTC), person-centred approaches are encouraged. One such approach, relationship-based care (RBC), aims among other things to reduce residents' agitated behaviours. RBC has been used in numerous Quebec LTC facilities over the past decade but it has never been studied. *Objective*. Explore correlations between use of RBC by trained caregivers and the frequency of agitated and positive behaviours of residents with cognitive impairments. *Methods*. Two independent raters observed fourteen caregiver/resident dyads in two LTC facilities during assistance with hygiene and dressing. Checklists were used to quantify caregivers' RBC use and residents' agitated and positive behaviours. *Results*. Scores for RBC use were high, suggesting good application of the approach by caregivers. Correlation analyses showed that offering residents realistic choices and talking to them during care were associated with both positive and agitated behaviours (*P* from 0.03 to 0.003). However, many other components of RBC were not associated with residents' behaviours during care. *Conclusions*. There were only a few quantitative links between the RBC checklist items and the frequency of agitated or positive behaviours. Other studies with a more rigorous research design are needed to better understand the impact of relationship-based care on residents' behaviours.

## 1. Introduction

### 1.1. Person-Centred Care: Foundations, Implementation, and Effects

In Quebec, the mission of residential long-term care (LTC) centres is to provide quality care to clients who are severely impaired physically and especially cognitively [1]. Because of cognitive impairments, residents in these centres frequently display problem behaviours [2–4]. These behaviours affect not only the well-being of their formal caregivers (e.g., long-term care staff) [5–7] but also the residents' own quality of life [8]. 

To meet the needs of clients who present problem or agitated behaviours, new approaches have been developed over the years. Person-centred care is designed to be an alternative to or to complement pharmacotherapy in reducing problem behaviours in individuals with dementia [9]. According to the Committee on Quality of Health Care in America, person-centred care is one of the main areas for improvement that the health care system should address in order to increase the quality of health care, especially long-term care [10]. Such approaches are based on a humanistic concept of health care, where the primary focus must be on the person and his/her life experience and capacities, rather than characterising the person solely by his/her disease [11]. Person-centred care is the opposite of task-centred care. In long-term care, this principle is operationalised in an array of practices aimed at helping residents to establish relationships, be treated as persons with their own life history and interests, and live in an environment that resembles a living environment [12]. From the perspective of long-term care, this conception entails a set of practices aimed at helping the person enter into a relationship (with formal and informal caregivers and other residents) (being in a relationship) and be seen as having a life history and his/her own interests (being in a social world) [11]. The person-centred care approach also implies a favourable context, particularly in terms of the organization of the nursing staff's work (being in place), and a desire to respect the values and preferences of persons when providing care (being with self) [12].

Implementation of person-centred care (PCC) depends not only on the caregivers acquiring skills and knowledge but also on adapting the entire care context (care practices, work organisation, and physical environment) to tailor it to both residents' and caregivers' needs and preferences. This means that there must be flexibility in the organisation, meals, hygiene, and dressing assistance, and so on [13, 14]. Finally, the physical environment must also be adapted to the perspective that it is both a living environment for the residents and a workplace for the caregivers [15].

The results of the research on the effects of implementing PCC are not unanimous about reducing problem behaviours (such as wandering, aggression, and being noisy) or improving well-being during care of residents in long-term care. In 1999, Opie and colleagues [16] conducted a systematic review of studies published in the preceding decade on nonpharmacological strategies to reduce residents' problem behaviours. Despite the methodological limitations of the studies reviewed, 27 of which were described as poor, these researchers concluded that various strategies were effective, including caregiver training and environmental modifications. The literature review published a few years later by Landreville and colleagues [17] reached a similar conclusion: the authors even suggested that caregiver training and environmental modifications are the most effective approaches according to the studies reviewed, the majority of which were quasi-experimental. More recently, however, the meta-analysis done by Kong and colleagues in 2009 [18], which included only randomised clinical trials, maintained that only one nonpharmacological approach (sensory intervention) helped to significantly reduce problem behaviours in dementia. Caregiver training, environmental modifications, and the use of activities, among other things, did not seem to produce any positive outcomes in that regard.

### 1.2. Context of the Study

One of these approaches was developed in Quebec by the *Association pour la Santé et la Sécurité du Travail, Secteur des Affaires Sociales* (ASSTSAS; Association for Occupational Health and Safety in the Social Affairs Sector). The approach is called *relationship-based care *(RBC) and its objective is to improve both care for residents and occupational health for caregivers. It comprises an array of care practices that help to maintain residents' mobility and function as long as possible. Another aim and expectation of implementing this approach is a reduction in residents' agitated behaviours.

Relationship-based care (RBC) was developed from a French approach called manutention relationnelle (relationship handling) of Gineste and Pellissier [19] and is based on training tested in and adapted to the situation in Quebec. The goal of the training is not only to acquire skills and knowledge (how to approach residents, interpret their feedback and react in relationship mode, stimulate optimal autonomy based on realistic expectations, and gently ease contractures), but also to develop and maintain humanistic attitudes despite difficulties and constraints.

RBC training is given mostly to patient attendants (orderlies) and, to a lesser extent, other types of workers: nurses, nursing assistants, occupational therapists, physiotherapists, and recreation technicians. The main elements advocated in relationship-based care are outlined in Table 3.

Implementation of RBC in an institution starts with designating a project leader in the institution. Step 1 is a two-day basic group training session for about a dozen caregivers. The next step is a half-day individual session with a peer coach during which each participant delivers two types of care under the supervision of the instructor, who provides personalised feedback. The final step is a half-day consolidation session approximately one month after the training, which brings together all the trainees with their immediate supervisors to review the entire approach and discuss implementation challenges.

### 1.3. Aim of the Study

Although it has been used in many long-term care institutions in Quebec since 2002, relationship-based care has never been studied with residents or caregivers. The present study focussed specifically on residents. The aim was to explore correlations between Relationship-Based Care (RBC) and the positive and agitation behaviours of residents during assistance with hygiene and dressing. These types of care are some of the interventions with long-term care residents, including individuals with dementia, that trigger the most problem or agitated behaviours since it is impossible at such times to avoid entering their personal space [4]. The working hypothesis was that greater use of RBC by caregivers trained in this approach would be negatively correlated with the frequency of agitated behaviours and positively correlated with the frequency of positive behaviours of residents with cognitive impairment in long-term care facilities.

## 2. Methods

This exploratory study is part of a larger research project examining the impact of Relationship-Based Care on caregivers and institutions engaged in implementing this approach. The decision to use a correlational cross-sectional observational design for this initial study of RBC and residents was based on practical as well as ethical considerations, which prevented the use of an experimental design. It was both ethically and practically impossible to stop using this humanistic approach with some of the patients since RBC was the foundation of patient care.

### 2.1. Participants

The participating residents and caregivers were recruited in two Quebec LTC facilities where RBC had been used for a few years. To be eligible for the study, *residents* had to meet the following criteria: (1) have been living in the long-term care facility for at least three months; (2) have presented at least one instance of agitated behaviour or resistance to care in the previous week or presented a clinical profile in which mental impairment or mixed impairment (mental and physical) was predominant. *Caregivers* had to be patient attendants (orderlies) or nursing assistants who had been trained in RBC.

### 2.2. Data Collection

This study was approved by the Research Ethics Committee of the Centre de Santé et de Services Sociaux, Institut Universitaire de Gériatrie de Sherbrooke (approval number MP-IUGS-09-08). Consent forms were signed by caregivers. Since the majority of the residents were unable to provide informed consent, written consent for them to participate in the study was obtained from their legal representatives. The caregivers also asked residents for their verbal consent before starting the observations. We observed the two members of the dyad (caregiver/resident) during assistance with hygiene and dressing in the early morning or evening, simultaneously by two trained independent observers without any connection to the institution. The observers had received theoretical training on the measuring instruments before administering them to the residents (pre-tests) with a member of the research team. The results were compared. Any differences were discussed and explained. The frequency of the caregiver's actions, attitudes and behaviours expected when using RBC was scored by one of the two observers using a checklist specifically developed for that purpose (see below). At the same time, the frequency of the resident's positive and agitated behaviours during care was quantified using two instruments (see below) by the other observer. Both observers were in the room but stayed out of the way as much as possible to avoid disturbing the proceedings while being able to observe as well as possible.

### 2.3. Measuring Instruments

The degree of use of relationship-based care was estimated using the *RBC* Use Checklist. This checklist was initially developed by a member of the research team from (1) components used by ASSTSAS for the instructor's evaluation and feedback during the peer coaching step and (2) an in-house tool from a Quebec LTC facility. The checklist items generated were discussed by the research team, then validated and commented on by ASSTSAS advisors, whose recommendations were incorporated in the checklist. The ASSTSAS advisors are the individuals who developed the RBC training and who trained instructors in the workplace. Finally, a pretest by a team member led to additional modifications to make the instrument realistic and usable in the context of this study. The final checklist consisted of 23 items divided into five categories: *making contact* (6 items), *relationship bubble* (8 items), *general approach* (5 items), *teamwork* (2 items), and *communication* (2 items) (see Table 3). The observer had to indicate how often the behaviours expected from the caregiver occurred during care. A score was assigned to each item, that is, present (1) or absent (0), for the six items in the *Making contact* category, while a four-level score, that is, always present (3), generally present (2), rarely present (1), or absent (0), was assigned to the items in the other categories. The higher the score is, the better the RBC was correctly used. The observers could also indicate “not applicable” or “not observable.”

The resident's behaviours during care were estimated using two instruments, one for agitated behaviours and the other for “positive” behaviours. The frequency of occurrences of physical and verbal agitation during care was quantified using the Cohen-Mansfield Agitation Inventory (CMAI) [20]. The rater uses the inventory to note the frequency of 29 agitated behaviours in real time. At a given time, agitated behaviours are rated on a seven-point scale from 0 (behaviour not observed) to 6 (behaviour observed constantly). A higher score means more agitation of the resident during the care. The internal consistency of the *CMAI* (*α* = 0.86, 0.91, and 0.87 for daytime, evening and nighttime) and its interrater (*r* = 0.82) and test-retest (*r* = 0.83; *P* < 0.001) reliability can be considered good [21–23]. We estimated observable positive behaviours using an instrument developed by the research team and ASSTSAS advisors, called the Positive Behaviour Inventory (PBI), a tool derived from the Geriatric Indices of Positive Behavior [24]. The PBI contains 14 items including 5 verbal and 9 nonverbal indicators, with the same scoring scale as the CMAI. Verbal indicators were as follows: tries to communicate, participates in conversation, initiates conversation, asks to participate actively in own care and thanks the caregiver. Nonverbal indicators were: opens eyes, does not resist care, relaxes muscles, shows affection (e.g., caresses caregiver's arms), makes eye contact, makes appropriate movements during care, smiles, participates in care to the best of his/her ability, and shakes hands. A higher score indicates more frequent positive behaviours during the care. The test-retest reliability of the original version of the Geriatric Indices of Positive Behavior is good (*k* = 0.80) but the reliability of the PBI has not been studied.

The residents' *sociodemographic characteristics* and the caregiver' sociodemographic variables were also collected. In addition, the residents' functional autonomy was measured with the Functional Autonomy Measurement System (SMAF), which is used to estimate functioning in five dimensions: activities of daily living (ADL) (7 items), mobility (6 items), communication (3 items), mental functions (5 items), and instrumental activities of daily living (IADL) (8 items) [25]. In long-term care facilities, IADL are not systematically assessed and were not considered here. The score for each dimension is obtained by adding the item scores, which range from 0 to 3; a higher score indicates a high degree of dependence (maximum of 63). A reliability study showed that the intraclass correlation coefficient for total SMAF scores was 0.95 (95% confidence interval (CI): 0.90 to 0.97) for test-retest and 0.96 (95% CI: 0.93 to 0.98) for interrater reliability [26].

In addition, after-care, caregivers were asked about their perception of their application of RBC during the observed care. We asked them to express their perception as a percentage, with 100% being care that totally adhered to RBC principles as taught during the training and 0% being care that was completely inconsistent with RBC principles. In addition, they were asked about (1) their satisfaction with their care, (2) how they felt during the care (caregiver's feelings), and (3) how they thought the resident felt. For these three questions, the measuring scale consisted of five faces with the expression of the mouth on a continuum from very sad (inverted smile) to very happy (big smile). The caregivers had to indicate which of the faces reflected the situation in question. For each face, a score from −2 to +2 was assigned.

### 2.4. Data Analyses

The participants' (residents and caregivers) characteristics and the scores obtained on the measuring instruments were first described by mean and standard deviation or frequency and percentage, depending on whether the variable was continuous or categorical. Since some resident/caregiver dyads were observed more than once during assistance with hygiene and dressing, the mean of the scores for each resident was calculated and used for the analyses.

To achieve our objective, correlation analyses, controlled for duration of care, linked the caregivers' scores obtained on each of the RBC checklist items with the scores obtained on the instruments observing residents' behaviours. We also examined correlations between the caregivers' perceptions after-care, the residents' behaviours, and use of RBC (score on the *RBC Use Checklist*).

## 3. Results

### 3.1. Participants' Characteristics

A total of 14 residents and 6 caregivers participated in the study.  Table 1 presents the residents' characteristics. Some data are missing for two residents from one of the facilities (died shortly after the observations). As expected, the residents were very dependent functionally and their mental functions were very impaired. The majority of the caregivers were women, patient attendants (orderlies), all working full time, mostly on the day shift, with many years of work experience in the institution or on the patient care unit (Table 2). They had all been trained on RBC, some more recently than others, nearly two years before on average.

### 3.2. Use of RBC by Caregivers

Table 3 presents the mean scores for RBC use, by category and item. On average, the scores suggest that RBC items were applied by the caregivers most of the time when necessary (mean score for checklist items was 2.6 out of a maximum of 3: mean score of 86.6%). In the *Making contact* category, where the rating is based on the presence or absence of the item, only the “touches the resident” item was done less (mean score of 0.66/1). The other desirable actions when initiating care were done most of the time. For the items referring to the relationship with the resident during care (*Relationship bubble*), it was found that in general caregivers looked at the residents, spoke to them, told them what they were going to do, and touched them gently. Massage was rarely used (*n* = 3) since, in RBC, it is recommended for bedridden clients with muscle contractures. Caregivers obtained very high scores for items in the *General approach*, except for “ends the care.” *Teamwork* did not often come into play but, when used, was used as expected in RBC. Finally, *Communication* with residents was good (mean scores between 2.71 and 2.90/3).

Following-care, the caregivers' self-rated percentage of application of RBC was high (mean 86%; SD 11.0) (data not shown). Their mean satisfaction can be considered positive (1.38; SD 0.43; maximum score 2: 69%) and they felt good during the care (1.56; SD 0.39; 75%). Caregivers' perception of how residents felt during care was quite positive but not as good as their perception of their own feelings (mean 1.24; SD 0.62; 62%).

### 3.3. Residents' Behaviours

The residents' scores on the instruments used to observe agitated and positive behaviours are presented in Table 4. Positive behaviours were observed more often than agitated behaviours.

The agitated behaviours observed most often during care were negativity, complaining, grabbing, and screaming. Despite a mean score close to 5, it is important to note that the frequency of agitated behaviours varied greatly from one resident to the next (SD 6.5). Sometimes agitated behaviours were rarely observed and although in other cases they were observed at various times during care, the mean duration of care was only 16 minutes, which accounts for their relatively low number.

The positive behaviours observed most often were participating to the best of their ability, making eye contact, and not resisting care. Relatively speaking, positive nonverbal behaviours were observed more often than verbal behaviours.

### 3.4. Correlations between RBC Use and Residents' Behaviours

Residents' agitated behaviours were significantly and negatively correlated with two RBC checklist items: (1) speaks to the resident during care (*r* = − 0.75; *P* = 0.003) and (2) offers realistic choices (*r* = − 0.62; *P* = 0.03). Both of these items are in the *Relationship bubble *category.

Residents' positive verbal behaviours were associated with two RBC checklist items: (1) touches the resident when making contact (*r* = − 0.70; *P* = 0.008) and (2) offers realistic choices (*r* = 0.59; *P* = 0.036). It is important to note here that the correlation between the presence of touching when making contact and residents' positive verbal behaviours was negative. Positive nonverbal behaviours were also associated with the same two items in the *Relationship bubble* category: (1) speaks to the resident during care (*r* = 0.76; *P* = 0.003), and (2) offers realistic choices (*r* = 0.67; *P* = 0.012). 

### 3.5. Correlations between Caregivers' Perceptions After-Care and Residents' Behaviours

No significant relationship was found between, on the one hand, the caregivers' perception of the percentage application of RBC and, on the other, RBC use observed by the rater or the residents' behaviours. No significant relationship was found either between the caregivers' perception of the percentage of application of RBC and their satisfaction with the care. However, statistically significant correlations were found between caregivers' feelings about the care they gave and residents' positive verbal (*r* = 0.57; *P* = 0.04) and nonverbal (*r* = 0.59; *P* = 0.03) behaviours. Similarly, caregivers' perception of residents' feelings was associated with positive verbal (*r* = 0.75; *P* = 0.004) and nonverbal (0.75; *P* = 0.003) behaviours.

## 4. Discussion

The objective of this study was to explore for the first time correlations between RBC use and residents' positive and agitated behaviours during assistance with hygiene and dressing. First, the data from the observations of the caregivers show that, generally, RBC items were applied by the caregivers quite often. Second, the data from observations of the residents suggest that agitated behaviours were present but varied from one resident to the next. Positive behaviours were more frequent, particularly nonverbal behaviours, than problem or agitated behaviours. Finally, the correlation analyses show few significant associations between the residents' behaviours and the frequency of use of RBC checklist items.

### 4.1. Use of Relationship-Based Care

In general, based on external independent observations, caregivers apply RBC well throughout the care, for a general mean use rate of 86.6%. This high percentage of application of the approach is comparable to those obtained in previous studies. For example, in the study by Bourgeois and colleagues [27], the mean of application of communication techniques similar to those encouraged in RBC varied from 50 to 95%, according to the behaviours. Following the training based on a person-centred approach, participants in Hoeffer and colleagues' study [7] applied gentleness during bathing (e.g., spoke quietly) for a percentage of 82.8 to 85.5%. However, in Drach-Zahavy's study [28], the score obtained for their participants' use of patient-centred care was lower (2.08/3), for an application rate of 69.3%.

Even though Epstein and colleagues [29] considered that using measures that are based on caregivers' perceptions to estimate their level of application of a patient-centred approach may be biased, it is very interesting to note that when asked about their personal perception of RBC application, caregivers gave themselves a mean score of 86.3%, which is very similar to the mean rate obtained by the external observer (86.6%). These virtually identical assessments from two different sources support the validity of the RBC checklist developed from various sources. These scores suggest that caregivers are very familiar with RBC and are very aware of whether they are applying it or not. Therefore, the use of caregivers' perceptions might be not so biased. This would be an interesting topic for future research since caregivers' perception could be less complex and less expensive than using external observers.

Although caregivers gave themselves a high score for RBC use, their satisfaction with their care was acceptable but not optimal (mean score of 69%). Nevertheless, they generally felt good during care (mean score of 75%), which was also found in other studies. In the study by Coen and colleagues [30], with a single-group before-after intervention design, even though there was no impact on caregivers' quality of life, burden, or well-being, their satisfaction increased after an education and support program about dementia care. Similarly, the perception of confidence and ease in giving care among Hoeffer's participants [7] increased after training. In another part of our research project carried out with 420 caregivers trained in RBC [31], job satisfaction was among the best perceived positive effects of RBC. However, Boumans and colleagues [32] found that only 3 out of 15 items related to quality of life at work significantly differed between caregivers who applied patient-oriented care after training and a control group.

Satisfaction with care score was significantly associated with residents' positive verbal and nonverbal behaviours but not with problem behaviours during care. Our exploratory cross-sectional study design did not allow us to establish a causal connection but we could hypothesise that the presence of positive behaviours by the residents might have a greater impact on the relationship with caregivers than problem behaviours. Thus, despite the presence of agitated behaviours, when residents present positive behaviours, such as making eye contact, smiling, or participating in their care to the best of their ability, the caregiver can feel good during the care.

### 4.2. Correlation between RBC Use and Residents' Behaviours

Only a few correlations were identified between RBC use according to the checklist and residents' behaviours. Generally, in our study, applying RBC more or less was not associated with greater or less frequency of residents' behaviours. It is important to reiterate that overall RBC was applied well by the caregivers, which reduces the variability needed to establish significant correlations. However, two items in the *Relationship bubble* category of the RBC Use Checklist, namely speaking to residents and offering them realistic choices, were found to be related to residents' problem as well as positive behaviours. Thus we could hypothesise that the more caregivers speak to residents and the more they allow them to make choices, the more the residents present positive behaviours. Caregivers who participated in the qualitative study by Skovdahl and colleagues [33] noted the importance of empowering residents to make decisions in preventing the occurrence of problem behaviours. However, it is also possible that it was the residents' positive behaviours that induced the caregivers to talk to them more and offer them choices during care. Our research design allowed us only to make a determination regarding the presence of a positive correlation between these variables, not to establish a causal connection.

In RBC, making initial contact with residents is important. One of the elements advocated to establish contact is to touch the resident physically at the beginning of the relationship. A statistically significant correlation was found between the presence of touching when making contact and residents' positive behaviours. According to RBC, this touching helps to reduce agitated behaviours but our study was unable to confirm this correlation. However, our data suggest that touching at the beginning of care is associated with fewer positive verbal behaviours during care. This result is difficult to explain and may be a fluke.

To summarize, the majority of RBC checklist items were not statistically associated with residents' agitated or positive behaviours as measured in this study. These results are similar to those of some studies and contrary to others. Some studies without a control group, like that by Mathews and colleagues [34], concluded that residents' verbal agitation was reduced when implementing a person-centred approach. Similarly, Mickus and colleagues [35], following a short interactive training session with nurses, observed a lessening in the frequency of residents' problem behaviours. Also, some systematic reviews [17, 18] suggest that person-centred approaches are effective in reducing agitated behaviours. However, the meta-analysis of randomised clinical trials by Kong and colleagues [18] showed that those approaches were not effective in that regard. Overall, there is no consensus or conclusive evidence regarding the impact of using person-centred approaches with clients with cognitive impairments on reducing problem behaviours. For example, in their randomised clinical trial with persons with dementia, Beck and colleagues [36] concluded that their person-centred approach did not help to reduce problem behaviours. On the other hand, training certified nurses to use person-centred strategies helped to reduce aggression and agitation in the participants in a study by Sloane and colleagues [37] using a randomised crossover design.

### 4.3. Strengths and Limitations

The limitations of this exploratory study should be noted. Because of the cross-sectional observational design, it was impossible to establish causal connections or determine the effectiveness of RBC. The sample size was not optimal because in Quebec Bill 21 considerably limits the participation of people with cognitive impairments in research and at the time of this study, only residents with legal representatives could participate in research. Also, the direct observations in residents' rooms could have disturbed the residents and affected the caregivers' work. It would have been interesting to videotape the care from different angles, which might have shed more light on the caregiver/resident relationship. Finally, the checklists used to estimate use of RBC and note positive behaviours were developed specifically for this study and, apart from content validity in their development process, their metrological properties have not been studied.

This study also has some significant strengths. To our knowledge, this is the first time residents' positive behaviours were taken into account and not only agitated behaviours. Also, the degree of use of RBC and occurrence of behaviours were rated by two independent observers not connected to the care facilities, one observer for the resident and the other for the caregiver.

## 5. Conclusion

In our exploratory study, the caregivers observed applied many of the elements advocated in RBC. As for the residents, problem behaviours were present during assistance with hygiene and dressing, which necessarily involves invading their privacy. Since there was no control group or pretraining measure, we cannot say if the residents' agitated behaviours would have been more frequent if the caregivers had not used RBC. However, we can clearly state that there were only a few quantitative links between the RBC checklist items and the frequency of agitated or positive behaviours.

The desired impact of RBC on reducing the frequency of agitated behaviours is not the only reason to implement this approach in long-term care facilities. In fact, RBC aims to provide quality care to residents, each of whom is unique, to ensure they live their last years in dignity despite disease, by meeting their needs as well as listening to the needs of the family and society and taking a humanistic approach to these individuals.

## Figures and Tables

**Table 1:** Residents' sociodemographic and clinical characteristics (*n* = 14).

Continuous variables	Mean (standard deviation)
Age (*n* = 12)	78.3 (14.4)
Functional autonomy (*n* = 12)	
SMAF ADL (/21)	16.5 (4.3)
SMAF mobility (/18)	8.8 (2.4)
SMAF communication (/9)	1.4 (1.2)
SMAF mental functions (/15)	10.1 (3.4)
SMAF total (/63)	36.8 (8.3)

Categorical variables	Frequency (%)

Sex	
Men	7 (50.0)
Women	7 (50.0)
Language	
French	9 (64.3)
English	4 (28.6)
Other	1 (7.1)
Marital status (*n* = 12)	
Married	2 (14.3)
Widowed	3 (21.4)
Never married	4 (28.6)
Separated/divorced	3 (21.4)

SMAF: functional autonomy measurement system.

ADL: activities of daily living.

**Table 2:** Caregivers' characteristics (*n* = 6).

Continuous variables	Mean (standard deviation)
Age	47.3 (3.4)
Years of experience in the institution	15.8 (6.5)
Years working on the patient care unit	10.5 (8.0)
Number of months since RBC training	22.5 (19.4)

Categorical variables	Frequency (%)

Position	
Patient attendant (orderly)	5 (83.3)
Nursing assistant	1 (16.7)
Sex	
Women	5 (83.3)
Men	1 (16.7)
Work shift	
Day	5 (83.3)
Evening	1 (16.7)

**Table 3:** Mean scores for RBC items obtained by the caregivers when assisting residents (*n* = 14 with some exceptions) with hygiene and dressing.

Items	Mean (standard deviation)
Making contact (score 0 or 1)	
(1) Knocks on the door (*n* = 10)*	0.81 (0.33)
(2) Introduces him-/herself (*n* = 13)*	1.0 (0)
(3) Announces what the care will be	0.90 (0.19)
(4) Looks at the resident	0.98 (0.07)
(5) Speaks to the resident	0.98 (0.07)
(6) Touches the resident	0.66 (0.36)
**Total of the RBC Making contact items (/6)**	**4.8 (0.7)**
Relationship bubble (/3)	
(7) Looks at the resident during care	2.73 (0.37)
(8) Announces what he/she will do	2.85 (0.35)
(9) Speaks to the resident during care	2.66 (0.79)
(10) Touches, moves the resident gently	2.67 (0.41)
(11) Maintains physical contact	2.02 (0.97)
(12) Announces if leaving	1.94 (0.99)
(13) Uses massage (*n* = 3)*	1.20 (1.69)
(14) Offers realistic choices	2.24 (0.86)
**Mean of the RBC Relationship bubble items (/3)**	**2.5 (0.5)**
General approach (/3)	
(15) Adapts interventions to feedback	2.53 (0.71)
(16) Ensures comfort (physical and mental)	2.81 (0.33)
(17) Asks resident to participate, allows autonomy	2.84 (0.30)
(18) Prefers standing during care	2.97 (0.09)
(19) Ends the care	1.91 (1.05)
**Mean of the RBC General approach items (/3)**	**2.6 (0.3)**
Teamwork (/3) (*n* = 5)*	
(20) Does not speak at same time as coworker	2.70 (0.67)
(21) Is client- and task-oriented	2.29 (0.65)
**Mean of the RBC Teamwork items (/3)**	**2.3 (0.5)**
Communication (/3)	
(22) Gives clear instructions	2.90 (0.27)
(23) Suggests positive ideas/positive reinforcement	2.71 (0.57)
**Mean of the RBC Communication items (/3)**	**2.8 (0.3)**

*indicates that these items were observed during the care of only some, not all 14, of the residents.

**Table 4:** Residents' behaviours during care and mean duration of care.

	Mean (standard deviation)
Agitated behaviours	
Cohen-Mansfield Agitation Inventory (/72)	4.9 (6.5)
Positive behaviours	
Verbal (/30)	6.6 (4.8)
Nonverbal (/54)	20.7 (7.3)
Duration of care (minutes)	16.2 (6.0)
